# Auxological and Endocrinological Features in Children With McCune Albright Syndrome: A Review

**DOI:** 10.3389/fendo.2020.00522

**Published:** 2020-08-04

**Authors:** Maria Tufano, Daniele Ciofi, Antonella Amendolea, Stefano Stagi

**Affiliations:** ^1^Pediatric Unit, Mugello Hospital, Borgo San Lorenzo, Florence, Italy; ^2^Health Sciences Department, University of Florence, Anna Meyer Children's University Hospital, Florence, Italy; ^3^Pediatric Unit, Cecina Hospital, Livorno, Italy

**Keywords:** McCune–Albright syndrome, fibrous dysplasia of bone, *café au lait* skin pigmentation, endocrinopathies, precocious puberty

## Abstract

McCune–Albright syndrome is a rare and challenging congenital sporadic disease involving the skin and skeletal and endocrine systems with a prevalence ranges from one in 100,000 to 1,000,000. In addition to the classical triad of fibrous dysplasia of bone, *café au lait* pigmented skin lesions and precocious puberty, other multiple endocrinological features, including hyperthyroidism, growth hormone excess, hypercortisolism, and hypophosphatemic rickets, have been reported. A brief review of the syndrome in children is here reported.

## Introduction

McCune–Albright syndrome (MAS, OMIM #174800), first reported nearly 80 years ago by McCune (1) and by Albright (2), is a rare and challenging congenital sporadic disease involving the skin and skeletal and endocrine systems (3). The estimated prevalence of MAS ranges from one in 100,000 to 1,000,000, involving both females and males with no differences across ethnic groups (3).

Initially, MAS was defined as a triad of mono/polyostotic fibrous dysplasia (FD) of bone, *café au lait* pigmented skin lesions (CALMs), and precocious puberty (PP) (3), even if other multiple endocrinological features, including hyperthyroidism, growth hormone (GH) excess, hypercortisolism, and hypophosphatemic rickets, have been reported (4–6). MAS may also include various other disorders such as hepatic and cardiac involvement (6).

FD is the most frequent feature in MAS and it is often accompanied by extraskeletal manifestations (3, 6). Only a small percentage of cases manifests in the absence of any FD (6, 7). Diagnosis of MAS is most often established in early childhood, but it may occur at birth due to the presence of CALMs (6).

## Genetic Basis

MAS is due to somatic postzygotic activating mutations in the *GNAS* gene (*Guanine Nucleotide-Binding Protein, Alpha-Stimulating Activity Polypeptide*) located on the long arm of chromosome 20 (20q13.3) (4, 8, 9).

*GNAS*, encodes the ubiquitously expressed stimulatory G-protein alpha subunit (Gsα) that regulates the synthesis of cyclic adenosine monophosphate (3, 8–11). The most common *GNAS* mutations recorded in MAS involve to the amino acid substitutions at Arg 201 (to Cys or His or Ser or Gly) and rarely at Gln 227 (to Arg or Lys) (about 5% of cases) (12). These mutations cause an increased Gsα protein signaling and consequently an increased function of glycoprotein hormone receptors, autonomous cell proliferation and increased hormonal secretion (8–11). In bone Gsα activation leads to increase of bone marrow stromal cells with inability to differentiate toward mature osteoblasts, adipocytes and stroma. This consequently leads to fibro-osseous tissue lacking of haemopoietic marrow (12).

Constitutive activation of the *GNAS* gene in the germline is probably responsible for early embryonic lethality. This phenomenon could explain the sporadic presentation of MAS. So each patient shows a *de novo* defect with a variable percentage of mutation in multiple affected tissues (11, 13). The final phenotype and the severity of disease depend on the developmental stage at which the mutation occurs and the type of affected cell lineages. So mutations at the inner cell mass stage result in involvement of multiple tissues of endodermal, mesodermal, and ectodermal origin (3, 10, 11).

Genetic testing on leukocyte DNA is possible, but it is often unreliable. The somatic mosaic nature of the disease frequently leads to false-negative results with an underestimation of causative molecular alterations, and the analysis of biopsies is often needed to obtain a molecular diagnosis. Recently, new highly sensitive techniques have shown to be able to improve *GNAS* mutant allele detection, especially in the blood (3, 14, 15).

### Skeletal Features

The characteristic skeletal features of MAS is FD (3, 11, 12), in which bone appears to be replaced and distorted by disorganized fibrous tissue (11, 16). The craniofacial bones, femur and pelvic bones are the most frequently affected sites (16, 17).

FD lesions, especially in the craniofacial region, begin to appear during pediatric period with the most of lesions present before the age of 15 years (16–18). In a study by Hart et al. (18), the majority of skeletal disease has been identified between 3 and 10 years of age. More than 50% of the new lesions established before the age of 10 years while 18% occurred after the age of 20. Concerning new lesions, the axial skeleton is involved in about 50% of cases while the extremities in 47% and the craniofacial region only in 2% (18).

Clinically the bone lesions could be divided in quiescent (stable without growth), non-aggressive (slow growing) and aggressive (rapid growth) lesions (16, 17).

FD diagnosis can be radiologically made showing expansive lesions usually smooth and homogeneous and in addition not centrally located within medullary bone, described as “ground glass” appearance. Craniofacial lesions typically demonstrated dense and sclerotic features (16, 17).

Computed tomography imaging better delineates morphological changes in bones and is considered the gold standard for radiological evaluations for the skull, while radiographs are indicated for evaluation of long bones (16, 17).

Magnetic Resonance could show typical signal intensity and enhancement in active lesions but it cannot discriminate FD from other entities (16, 17, 19). On the other hand it could be necessary for the evaluation of challenging cases of FD, such as in patients with compressions of neurological structures and in suspicion of aneurysmal bone cyst (16), a benign, tumor-like bone lesion composed of multiple cystic blood-filled compartments, exceptionally associated with MAS (20, 21).

Technetium 99m-methyl diphosphonate bone scan can be used to detect metabolically active lesions and to evaluate the extension of the disease, even if in younger children, in particular under 6 years, small areas of involvement could not be identified (16, 18). Biopsy is indicated for histological confirmation when typical radiographic features are lacking (17).

The craniofacial region is the most involved location and facial deformity or asymmetry, caused by an expanding FD lesion, can occur. Vision and/or hearing impairment, nasal congestion, pain, paraesthesia, and/or dental malocclusion may be the presenting symptoms (16, 17). The overall enlargement and deformity can appear severe and disfiguring, especially in patients with poorly controlled GH excess, even if a slowing down of the lesions may be observed after puberty (16–18).

The femur is the other common location of FD. The classical bone deformity described as the “shepherd's crook” deformity could present in different patterns according to the neck-shaft angle measurement and the presence or absence of lateral bowing of the proximal femoral shift (16).

Recurrent fractures, occurring especially between ages of 6 and 10 years, pain and progressive deformity can lead to disability and represents the most important complications (16, 22, 23). More frequent and severe pain has been registered in adults than in children (16, 23).

Malignant transformation of FD lesions is rare even if in MAS patients malignant changes to osteosarcoma and chondrosarcome have been reported (16, 24–26).

The goals of orthopedic surgery are reducing fractures, stabilizing impending fractures and correcting deformities while physical and occupational therapy may improve mobility and function (16, 17). Medical treatment with intravenous bisphosphonates has shown encouraging results demonstrating to reduce pain, decrease fracture rate and bone turnover even if there is no evidence that they could improve bone quality (16, 22, 27, 28) (Table 1).

**Table 1:** Therapy of clinical manifestations in children with MAS.

**Clinical features**	**Pharmacological therapy**	**Other therapies**	**References**
Fibrous dysplasia	Intravenous bisphosphonates, analgesic therapy	Surgery, physical therapy, and occupational therapy	(16, 22, 27, 28)
Precocious puberty in girls with secondary activation of the HPG axis	Aromatase inhibitors (letrozole) Receptor blockers (tamoxifen) Pure estrogen receptor antagonist (fulvestrant) Anti-androgens	Laparoscopic cystectomy should be reserved in case of significant abdominal pain or when ovarian torsion is present	(29–32)
	GnRH analog therapy		
Precocious puberty in boys with secondary activation of the HPG axis	Androgen receptor blocker (spironolactone, flutamide or cyproterone acetate) Aromatase inhibitors (letrozole) Inhibitors of steroidogenesis (ketonazole)	Surgery (few data, conservative approach is recommended)	(30–35)
	GnRH analog therapy		
Thyroid disease	Thionamides (methimazole)	Surgery or ablation with radioactive iodine	(6, 36)
Hypophosphatemia	Phosphate and active vitamin D (calcitriol)		(6, 22)
Growth hormone excess	Somatostatin receptor ligand (octeotride) GH receptor antagonist (pegvisomant) Dopamine agonists, such as cabergoline or bromocriptine	Surgery or radiotherapy as last choice	(37–39)
Cushing's syndrome	Metyrapone Ketokonazole (potential liver toxicity)	Surgery	(6, 40)

### Dermatologic Features

The CALMs arise from the ectoderm and represent a clinical manifestation of the mosaic nature of *GNAS* mutations, resulting from a localized increase of melanogenesis and melanin transfer to keratinocytes (41–43). In fact, melanocytes within the pigmented patch, but not in the surrounding healthy skin, have been found to present the somatic mutation (44).

The CALMs usually appear after birth representing the first manifestation of the disease (3, 6), even if classic CALMs usually manifest during childhood (3, 43).

**Figure 1:**
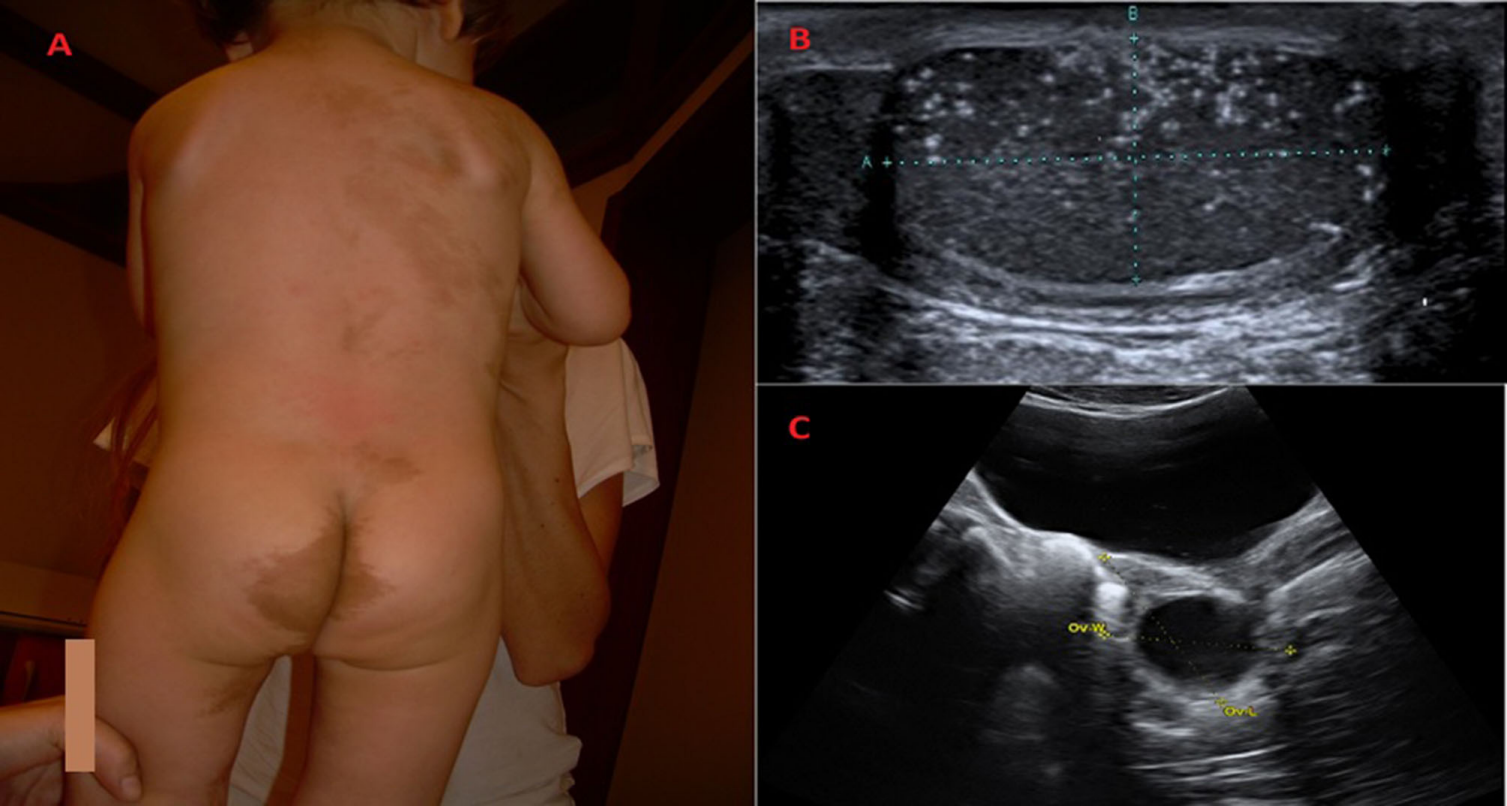
Clinical and radiological features of children diagnosed with McCune Albright syndrome. **(A)** Classic *Café-au-lait* skin pigmented lesion in a 25-months-old boy with McCune-Albright Syndrome. The spots with smooth borders, respect the midline and follow the developmental lines of Blaschko. At follow up, fibrous dysplasia of the both tibias has been revealed. **(B)** When he was 3-years-old, a light testicular asymmetry has been noted and testicular microlithiasis has been revealed on ultrasound. **(C)** Unilateral ovarian cyst in a 7-years-old girl with vaginal bleeding diagnosed with McCune Albright syndrome.

In comparison with the neurofibromatosis (NF) CALMs in MAS patients show typical clinical features, whereas histology cannot discriminate the lesions (42–44). As reported by Saggini et al. (42), MAS-related CALMs are usually fewer in number and larger in diameter, often with darker pigmentation and more irregular borders, on the contrary of NF, in which CALMs have smoother borders (Figure 1A). In MAS, CALMs usually do not go beyond the midline of the body and the developmental lines of Blaschko are followed (3, 42–44). Posterior neck, base of the spine, trunk, and face are common sites (42–44). No correlation between size and location of CALMs and severity of bone disease has been reported (6). Nevertheless, 10% of the healthy population can show CALMs (6, 45).

Oral melanotic pigmentation, a typical feature of several hamartomatous conditions such as Peutz-Jeghers syndrome, has also been described (46).

Effective treatments for the hyperpigmentation in MAS are not demonstrated while attempts to bleach hyperpigmented lesions usually leave an unsatisfying area of under pigmentation (6).

## Endocrinological Features

### Precocious Puberty

PP is one of the endocrinological features of MAS and, frequently, it is the clinical features suggesting the diagnosis (6, 29–31). PP is a form of peripheral rather than central PP since it is not mediated by hypothalamic- pituitary-gonadal (HPG) axis. Although girls and boys might theoretically be equally affected, a significantly higher prevalence of PP in girls than in boys has been reported (29–31). The clinical characteristics, the diagnosis and the treatment of PP are distinctly different in girls and in boys so it need to be considered separately (29, 30).

The typical presentation of PP in girls with MAS consists of sudden onset of painless vaginal bleeding, which may be either isolated or associated to the development of breast tissue and enhanced growth and bone maturation rate (6, 29, 30). Although it may be present during infancy, PP in girls with MAS usually becomes manifest during early childhood (6, 31). This represents the result of an intermittent autonomous activation of ovary leading to the formation of large ovarian cyst (6, 30). Elevated estradiol levels are typically revealed, sometimes many-fold higher than prepubertal values and 2-3 fold higher than levels achieved during menstrual cycle, associated to suppressed gonadotropins (29–31).

Pelvic ultrasound at the time of initial episode, will reveal asymmetry in ovarian volumes, since the cysts are usually unilateral (Figure 1C) (31). The cysts may be hemorrhagic and appear to have mixed cystic and solid elements. An enlarged uterus with an endometrial thickening with no evidence of ovarian cyst may also be noted (30, 31). The resolution of the cysts is followed by estrogen withdrawal with the subsequent vaginal bleeding and regression of uterine size (31). The cysts can be asymptomatic, so until the bleeding occurs, they could be unrecognized (6, 31).

An unpredictable natural history of PP in girls with MAS has been described with variable age at manifestation of the first episode ranging from the first few months of life, until 6 or 7 years (6). In the same way timing of following episodes are unpredictable with many girls having long periods of quiescence and others showing frequent episodes of vaginal bleeding associated to progressive sexual development (6). Linear growth acceleration and advanced bone maturation could also be observed (6, 31). In contrast to the described vaginal bleeding, some girls could manifest periodic variation of breast enlargement without bleeding (6).

Clinical management of PP in a girl with MAS includes clinical observation for girls with only sporadic and infrequent vaginal bleeding (6). In the subset of girls with a progressive form of PP, the primary goals of pharmacologic intervention are preventing vaginal bleeding and delaying the rate of bone age advancement, with the aim of improving adult height (6, 29–31). Several different pharmacological strategies have been utilized (29–31) (Table 1). Current treatment of PP in girls with MAS includes the use of anti-estrogens such as aromatase inhibitors, estrogen receptor blockers and pure estrogen receptor antagonist (fulvestrant) (29–31). In girls with secondary activation of the HPG axis, GnRH analog therapy has been reported to be beneficial as adjunctive therapy (30, 31). Surgery with laparoscopic cystectomy should be reserved in case of significant abdominal pain or when ovarian torsion is present (31, 32). Detailed comparison and update of the therapy of PP girls with MAS are above reported (29–31, 47).

PP in boys with MAS is very different from girls (6, 30, 31). The first difference is that PP is very rare in affected boys with MAS (6, 31, 32) The clinical presentation may include unilateral or bilateral testicular enlargement (due to autonomous hyperfunction and hyperplasia of Sertoli and Leydig cells), sexual precocity and pubertal serum testosterone levels (6, 30, 48, 49). Testicular autonomous hyperfuction in MAS may also be secondary to Sertoli cells activation with a consequent isolated macroorchidism usually not followed by signs of sexual precocity and prepubertal testosterone levels (30, 31). A higher incidence of testicular abnormalities on ultrasound (microlithiasis, hypoecoic lesions, focal calcifications) has also been described (33, 50, 51) (Figure 1B). None of the patients with these lesions, followed up for several years, developed any signs of malignant transformation, so a conservative approach has been recommended (33).

Therapy options for boys include combination of androgen receptor blocker such as spironolactone, flutamide or cyproterone acetate along in combination with drugs interfering with sex steroid synthesis such as ketoconazole or aromatase inhibitors (30–35) (Table 1). If progression to central PP occurred, addition of GnRH analog could diminish rate of skeletal advancement (30–32).

### Thyroid

Thyroid disease is the second most common endocrinopathy in patients with MAS with a prevalence reported of about 31%. The onset of thyroid disease ranged from 1 to 20 years (6, 36). The thyroid alterations can present as functional and/or morphological dysfunctions ranging from asymptomatic thyroid nodules detected on ultrasound to diffuse goiter to hyperthyroidism (6, 36). Although thyroid cancer has been described in few cases of MAS, the prevalence of malignancy does not appear to be high (52, 53). Indeed thyroid neoplastic lesions seemed to remain well-differentiated, while thyroid carcinoma most commonly express activating mutations of Gαs associated with other oncogene mutations (54).

The thyroid ultrasound findings in MAS include normal morphology, cystic or solid lesions, or macro/micronodular goiter (6, 55).

The prevalence reported of patients with symptomatic hyperthyroidism is variable. Hypertension, tachycardia and hyperactivity have been the most common symptoms reported (36, 56). Laboratoristic findings comprise suppressed thyroid stimulating hormone associated to elevated levels of triiodothyronine (T3); normal levels of thyroxine can be detected (6).

Hyperthyroidism in MAS could be treated adequately with pharmacological therapy (thionamides) (6, 35). Surgery or ablation with radioactive iodine represent the second choice therapy when hyperthyroidism cannot be adequately controlled with medications (6, 36, 56) (Table 1).

### Hypophosphatemia

Approximately half of patients with bone involvement presented with renal phosphate wasting (57). Overproduction of Fibroblast Growth Factor 23 (FGF23) could have a pathogenetic role (58).

FGF23 is a glycoprotein playing as an important factor in renal phosphate wasting (57). FGF23, acting in conjunction with parathyroid hormon, decreases phosphate reabsorption. This, in turn, results in hyperphosphaturia and hypophosphatemia. FGF23 is also a counter-regulatory hormone for 1,25(OH) vitamin D in the bone– kidney feedback loop (57). FGF23 is almost exclusively produced by osteocytes and osteoblasts in response to high serum phosphate levels and 1,25(OH) vitamin D and in FD bone tissue in patients with MAS (6, 57) So increased serum FGF23 levels appears to be related to the severity of bone disease. Therefore, significant hypophosphatemia is seen in patients with a very significant skeletal burden of FD (6, 57).

Elevated concentrations of FGF23 are responsible for impaired bone mineralization and so for more frequent fractures even if it is not simple to discriminate disease burden effects from phosphaturia/hypophosphatemia effects, because of correlation between FGF-23 levels and disease burden (6, 22, 57, 58).

Beneficial effect on the skeletal disease derived from correction of hypophosphatemia, with the use of phosphate and active vitamin D, has not been demonstrated (6, 22) (Table 1).

### Growth Hormone Excess

GH excess had a prevalence reported of 10–20% in MAS patients, usually associated to craniofacial involvement (6, 37, 38). Compared to classical acromegaly, the age at presentation and diagnosis is earlier (48.7 vs. 24.2 years) with a higher prevalence in males (37, 38).

Clinical features include enlarged feet and hands, facial asymmetry, visual, hearing or olfactory defects acromegalic cardiopathies (left ventricular hypertrophy, atrial or aortic widening and pericardial effusion), impaired glucose tolerance (6, 37–39). Among pediatric patients, the association with PP is frequent and in these cases accelerated growth should be an additional symptom (37–39). Furthermore hyperprolactinemia has been reported in the most of patients (6, 37). Pituitary adenomas (micro and macro adenoma) have also been reported (37, 38). Craniofacial dysplasia, in particular sphenoid bone involvement, is of clinical relevance in MAS patients with GH excess and usually associated to macrocephaly, vision loss and hearing deficit (6, 37–39). Higher concentration of GH accelerates craniofacial lesions with consequent raised risk of olfactory, hearing and visual deficits (37, 39).

The diagnosis of GH excess is usually based on laboratoristic findings revealing non-suppressible serum GH on an oral glucose tolerance test and high levels of GH and insulin-like growth factor 1 (IGF1) (6, 37).

The goal of treatment in children is to decrease the IGF-1 to the middle of the normal range while in adult patients is to decrease the serum IGF-1 to as low as possible (6). In MAS patients current treatment of GH excess includes radiotherapy, surgery and medical treatment (somatostatin receptor ligand, dopamine agonist- cabergoline- and GH receptor antagonist- pegvisomant) (37).

Use of a single drug generally does not provide sufficient control so better results have been obtained with combination therapy (cabergolin, a dopamine agonist, plus octreotide, octreotide plus pegvisomant) (37, 39) (Table 1).

Considering the risk of bone malignant transformation, radiotherapy is accepted to be as the last choice, to use when surgery is not possible and pharmacological therapies appear to be ineffective (6, 37).

The hyperprolactinemia, that is usually associated to GH excess, could be efficiently controlled with pharmacological therapy (dopamine agonists, such as cabergoline or bromocriptine) (6).

### Cushing's Syndrome

Cushing's syndrome is a rare endocrinological feature in patients MAS with a variable natural history, ranging from spontaneous resolution to need for surgical gland removal until death (6, 40). The poor prognosis of patients with Cushing syndrome has been secondary to associated heart disease and to opportunistic infections. Patients with significant cortisol excess could have secondary immunodeficiency so prophylaxis for Pneumocystis Carinii pneumonia should be considered in all patients before therapy (6, 40). Exposure to excess glucocorticoid, in utero or during early postnatal life, could result in a higher incidence of developmental problems among survivors (40).

Brown et al. (40) described a group of patients with MAS and Cushing's syndrome. The diagnosis is usually made in the first months of life as hypercortisolism is almost certainly present *in utero* in the most of patients (40, 59). The signs and symptoms reported were: small for gestational age, “Cushingoid facies,” failure to thrive with failure of both linear growth and weight gain, hypertension, nephrocalcinosis, hirsutism, hyperglycemia.

Cushing's syndrome in patients with MAS can resolve spontaneously even if it is not possible to predict in which patients this will occur (6, 40). Spontaneous resolution of hypercortisolism followed by residual autonomous adrenal function associated to adrenal reserve has also been descried (40).

Treatment of Cushing syndrome in MAS patients include medical treatment (metyrapone or other drugs) and surgical removal of adrenal glands (6, 40) (Table 1).

## Long-Term Health Problems

A wide spectrum of challenging health problems has been described in adult patients with MAS. Wong et al. (60) reported progressive facial asymmetry, T3-toxicosis, reduction in spermatogenesis. cardiological and lung complications, benign and aggressive tumors (gastrointestinal polyps, muscle myomas, breast cancer). Long-term cancer risk, even unknown, should require careful follow-up (24–26, 52–54). About the course of MAS in female during adolescence and young adult life, persistence of ovarian autonomy, due to estrogen hypersecretion, has been demonstrated. Significant effects on gynecologic function with abnormal uterine bleeding and on reproduction with an increased infertility prevalence have been reported (61–63).

Few data about the natural evolution of sexual development and the adult fertility prognosis in male patients can be recovered. According to available data, males seemed to be able to maintain their autonomous testicular function (33, 64).

## Conclusions

MAS syndrome is a rare and challenging disease with multisistemic involvement requiring multidisciplinary team of specialists for the treatment of manifestations and the surveillance of skeletal and extraskeletal complications.

## Author Contributions

MT wrote the manuscript with support from DC and AA. SS supervised the project.

## Conflict of Interest

The authors declare that the research was conducted in the absence of any commercial or financial relationships that could be construed as a potential conflict of interest. The reviewer MW declared a past co-authorship with one of the authors SS to the handling editor.
